# A novel selective spleen tyrosine kinase inhibitor SKI-O-703 (cevidoplenib) ameliorates lupus nephritis and serum-induced arthritis in murine models

**DOI:** 10.1093/cei/uxac096

**Published:** 2022-11-08

**Authors:** Somi Cho, Eunkyeong Jang, Taeyoung Yoon, Haejun Hwang, Jeehee Youn

**Affiliations:** Department of Biomedical Science, Graduate School of Biomedical Science and Engineering, Hanyang University, Seoul 04763, Korea; Department of Anatomy and Cell Biology, College of Medicine, Hanyang University, Seoul 04763, Korea; Department of Discovery Biology, Research Institute, Oscotec Inc., Seongnam-si, Gyeonggi-do 13488, Korea; Department of Discovery Biology, Research Institute, Oscotec Inc., Seongnam-si, Gyeonggi-do 13488, Korea; Department of Biomedical Science, Graduate School of Biomedical Science and Engineering, Hanyang University, Seoul 04763, Korea; Department of Anatomy and Cell Biology, College of Medicine, Hanyang University, Seoul 04763, Korea

**Keywords:** systemic lupus erythematosus, spleen tyrosine kinase, Syk inhibitor SKI-O-703, autoimmune disease

## Abstract

Spleen tyrosine kinase (Syk) plays a pivotal role in the activation of B cells and innate inflammatory cells by transducing immune receptor-triggered signals. Dysregulated activity of Syk is implicated in the development of antibody-mediated autoimmune diseases including systemic lupus erythematosus (SLE) and rheumatoid arthritis, but the effect of Syk inhibition on such diseases remains to be fully evaluated. We have developed a novel selective Syk inhibitor, SKI-O-592, and its orally bioavailable salt form, SKI-O-703 (cevidoplenib). To examine the efficacy of SKI-O-703 on the progression of SLE, New Zealand black/white mice at the autoimmunity-established phase were administrated orally with SKI-O-703 for 16 weeks. Levels of IgG autoantibody, proteinuria, and glomerulonephritis fell significantly, and this was associated with hypoactivation of follicular B cells via the germinal center. In a model of serum-transferred arthritis, SKI-O-703 significantly ameliorated synovitis, with fewer neutrophils and macrophages infiltrated into the synovial tissue. This effect was recapitulated when mice otherwise refractory to anti-TNF therapy were treated by TNF blockade combined with a suboptimal dose of SKI-O-703. These results demonstrate that the novel selective Syk inhibitor SKI-O-703 attenuates the progression of autoantibody-mediated autoimmune diseases by inhibiting both autoantibody-producing and autoantibody-sensing cells.

## Teaser

Orally administered SKI-O-703, a new Syk inhibitor, reduces lupus nephritis-like manifestations in NZB/W mice.

## Introduction

Systemic autoimmune diseases, such as systemic lupus erythematosus (SLE) and rheumatoid arthritis (RA), are characterized by activation of autoreactive T and B cells [1]. This activation represents a functional loss of self-tolerance and leads to autoantibody (autoAb) production. Hallmark autoAbs abundant in patients with SLE include antibodies (Abs) that recognize nuclear components such as histone, DNA, and ribonucleoproteins [2]. High titers of anti-double-stranded DNA (dsDNA) IgG Abs are specific for SLE and correlate with disease severity [3, 4]. Patients with RA also exhibit elevated levels of autoAbs, such as rheumatoid factors and anti-cyclic citrullinated peptide IgG Abs, although their pathogenicity is still a subject of debate [2]. Excessive amounts of such Abs are deposited into tissues as immune complexes and are sensed by innate inflammatory cells including macrophages and neutrophils via receptors recognizing their IgG Fc portions and complement. The inflammatory responses of these cells promote tissue destruction, further loss of self-tolerance, and, ultimately, the symptoms of autoimmune diseases [5]. Therefore, persistent production of pathogenic autoAbs and their sensing at the distal effector phase are the major disease-precipitating elements of the chronic systemic autoimmune diseases.

The pathogenic autoAbs enriched in SLE and RA are largely produced in reactions in germinal centers (GCs), in which antigen-primed follicular B cells undergo Ig gene class-switch recombination and somatic hypermutation during intensive proliferation, with the help with CD4^+^ follicular helper T (Tfh) cells [6–8]. Finally, a fraction of GC B cells gives rise to plasma cells secreting Ig class-switched and affinity-matured Abs. All these step-wise events are instructed by signals initiated by B cell receptor (BCR) cross-linking, which requires the activity of spleen tyrosine kinase (Syk).

Syk is a non-receptor protein tyrosine kinase acting as a key signal transducer downstream of BCRs [9]. BCR engagement with cognate antigens results in phosphorylation of tyrosine residues in the immunoreceptor tyrosine-based activation motifs (ITAMs) of Igα and Igβ chains within the BCR complex, which then recruit Syk via their SH2 domains. This event induces autophosphorylation and activation of Syk, leading to phosphorylation of direct downstream targets, such as SLP-65 and PLCγ1, and subsequent activation of diverse signaling cascades. Syk is required not only for the early development of B cells but also for the survival, proliferation, and differentiation of mature follicular B cells into GC B cells and Ab-secreting plasma cells [10–14]. For example, selective genetic ablation of Syk in primary B cells impairs T-dependent and -independent Ab responses *in vivo*, due to a severe reduction in GC B cells and plasma cells [12]. Thus, Syk appears to play an essential role in preserving Ab-dependent humoral immunity.

Apart from B cells, neutrophils and macrophages utilize Syk to transduce FcγR-triggered signals into the cell interior [15, 16]. FcγR cross-linking by IgG Ab on the surface of these cells causes phosphorylation of ITAM motifs of FcγR, which lead Syk to be recruited and activated. This signaling event favors the development of these cells to be phagocytic and pro-inflammatory.

There is evidence that Syk dysregulation is implicated in the pathogenesis of SLE and RA. Patients with lupus nephritis have infiltrates of Syk-expressing cells in their glomeruli [17]. The peripheral B cells of SLE patients with active disease exhibit increased levels of Syk phosphorylation [18, 19]. Conversely, genetic ablation of Syk in hematopoietic cells completely protected *Syk*^*−/−*^ bone marrow (BM) chimeric mice from K/BxN serum-transferred arthritis (KSTA), an experimental model of RA [20]. A cell-autonomous function of Syk in neutrophils may be involved in the development of KSTA since neutrophil-specific deletion of Syk protected the mice from this disease [21]. These investigations together have suggested that Syk is a potential target for treating Ab-mediated autoimmune diseases.

Compound R406 was initially reported to be a Syk inhibitor and its orally bioavailable prodrug, R788 (fostamatinib), was shown to ameliorate lupus-like disease in New Zealand black/white (NZB/W), MRL-lpr, and FcγRIIb-deficient C57BL/6 models of SLE, as well as serum-transferred and collagen-induced models of RA [22–26]. However, it turned out later that the inhibitor was non-selective, so it is unclear whether its activity was solely attributable to the inhibition of Syk activity [27]. Although fostamatinib is beneficial in suppressing autoimmune indications in clinical settings, its toxic effects appear to limit its usefulness [28, 29]. This has prompted us to seek a more selective Syk inhibitor to be able to draw unequivocal conclusions about the effect of Syk inhibition on such diseases.

We have synthesized a new Syk inhibitor named SKI-O-592 and its orally bioavailable salt form, SKI-O-703 (cevidoplenib). In the present study, we provide biochemical and *in vitro* evidence that this inhibitor is highly selective for Syk, targeting specifically BCR- and FcγR-mediated signaling events in B cells and innate inflammatory cells, respectively. Furthermore, oral administration of SKI-O-703 to NZB/W mice dose-dependently attenuated autoAb production and lupus nephritis-like manifestations. Optimal dose SKI-O-703 alone, or suboptimal dose SKI-O-703 combined with anti-TNF Ab, protected mice from KSTA by inhibiting Ab-mediated inflammatory responses. Thus, we demonstrate here that SKI-O-703 is a novel selective Syk inhibitor that targets two processes, namely B cell activation and innate inflammatory cell functioning, and consequently inhibits autoAb-mediated manifestations such as SLE and RA.

## Materials and methods

### Production of SKI-O-592 and -703

SKI-O-592, a novel pyrazolylpyrimidine compound, was synthesized and characterized by Oscotec Inc. (Seongnam-si, Korea), following a structure-based drug design approach. SKI-O-703 is a mesylate salt form of SKI-O-592 to be used orally in *in vivo* studies.

### Kinase assays

Kinase assays were conducted in 384-well plates using LANCE ultra 66 time-resolved fluorescence resonance energy transfer (TR-FRET) methods, according to the manufacturer’s instructions (Perkin-Elmer). In brief, each kinase was pre-incubated with a serially diluted compound for 30 min, incubated with ULight poly-GT peptide substrate and ATP for up to 1 h. Thirty minutes after adding Eu-labeled anti-phosphopeptides Ab diluted in LANCE Detection Buffer, LANCE signals were measured with an EnVision multilabel reader (Perkin-Elmer).

### Cell culture

THP-1 and Ramos cell lines and human CD14^+^ monocytes were purchased from the American Type Culture Collection (ATCC) and Lonza, respectively. After treatment with Syk inhibitors for 1 h, THP-1 cells and monocytes were stimulated for 15 min with 100 μg/ml human IgG (Invitrogen), and Ramos cells were stimulated with 2 μg/ml anti-human IgM monoclonal Ab (mAb) (Bathyl). Cells were lysed in RIPA buffer containing 1% NP-40, 1% sodium deoxycholate, 0.1% SDS, and a mixture of protease and phosphatase inhibitors (Thermo Scientific). The lysates were assayed by standard immunoblotting methods and ELISA. Primary Abs used for immunoblotting were anti-phospho-Syk (Y526/526), anti-phospho-Vav (Y174), anti-phospho-PLCγ1 (Y1217) and anti-phospho-BLNK (Y96) Abs (all from Cell Signaling). ELISAs to detect phospho-Syk (Y525/526) were performed according to the manufacturer’s protocol (Cell Signaling).

Human CD19^+^ B cells (Lonza) were pre-treated with inhibitors for 1 h and stimulated for 24 h with 50 nM CpG ODN 2006 plus 10 ng/ml IL-2 or 1 μM CpG ODN 2006 plus 20 ng/ml IFN-α. Culture supernatants were assayed by ELISA to detect secreted IgG, IgM, and IL-6, according to the manufacturer’s instructions.

Splenic CD19^+^ B cells from C57BL/6 mice were sorted by MACS (Milteny Biotec), stained with 3 μM cell proliferation dye eFluor 670 (CP670; eBioscience), and cultured for 72 h in the presence of either 10 μg/ml LPS (Sigma Aldrich) or 5 μg/ml anti-mouse IgM (Jackson Immuno Research Laboratories), 10 μg/ml anti-CD40 mAb (Biolegend) and 10 ng/ml IL-4 (Peprotech), and assayed by FACS. To detect apoptotic cells, unstimulated cells were cultured for 24 h, stained with Annexin V and 7-aminoactinomycin D (7-AAD), and assayed by FACS.

### Mice

Female NZB/W F1 mice were purchased from the Jackson Laboratory. C57BL/6 and BALB/c mice were purchased from Orient-Bio (Gyeonggi-do, Korea). K/BxN mice were bred in our facility by crossing KRN transgenic C57BL/6 mice with NOD mice [30]. All mice were maintained in a specific pathogen-free barrier facility at Hanyang University. The study was approved by the Institutional Animal Care and Use Committee (Approval numbers: 2020-0194, 2020-0184).

NZB/W female mice aged 18 weeks were assigned to four groups by distributing mice by body weight and anti-dsDNA IgG titer equivalently across the groups. Each group of mice was administered orally with 42 mg per kg body weight (mpk) of SKI-O-703 dissolved in 50 mM citric acid, 84 mpk of SKI-O-703, 30 mpk of tofacitinib (Selleckchem) dissolved in 0.5% carboxymethyl cellulose (Sigma Aldrich) containing 0.025% Tween 20, or a mixture of the two vehicles. The drugs were given once daily up to 34 weeks of age. Body weight was measured every 2 weeks and serum was collected every 4 weeks. At the termination of the experiments (34 weeks of age), serum, urine, spleen, BM, and kidneys were removed *post mortem* and assayed by histopathological and biochemical methods as below. Levels of blood urea nitrogen (BUN) and creatinine were measured by Knotus (Incheon, Korea).

Arthritogenic serum was collected from 8-to-15-week-old K/BxN mice. Eight-week-old male BALB/c mice were injected intraperitoneally (ip) with 100 μl of K/BxN serum to induce arthritis. The mice were treated orally twice daily with 42 or 84 mpk of SKI-O-703 from that day for 9 days. In some experiments, mice were injected ip every 2 days with 400 μg/mouse of the TNF blocker, etanercept (Amgen). Ankle thickness was measured, and arthritic index was determined daily, as described previously [31]. On day 9 post-induction, spleens and draining (inguinal and axillary) lymph nodes (LNs) were removed *post mortem* and assayed by histopathological and immunologic methods.

### Histopathologic examination

Kidneys removed from the NZB/W mice were fixed in 4% paraformaldehyde, embedded in paraffin, sectioned at 3 μm thickness, and stained with periodic acid-Schiff (PAS) and hematoxylin (Sigma Aldrich). Numbers of intra-glomerular cells per glomerular cross-section were counted, graded 0–3 (0 = 35–40 cells, 1 = 41–50 cells, 2 = 51–60 cells, 3 = > 60 cells), and displayed as glomerular hypercellularity [32]. Glomerular size was measured using Image J software. To assess the glomerular size and hypercellularity of each mouse, more than 20 glomeruli per mouse were individually examined and averaged.

Hind paws were removed from KSTA mice, fixed, and decalcified in 5.5% EDTA in phosphate-buffered formalin. The paws were embedded in paraffin, sectioned, and stained with hematoxylin and eosin (H&E). Arthritic changes in the ankle and foot were scored (0–4 scale) and expressed as a histopathologic index, as described previously [33]. Safranine O-stained sections were scored (0–4 scale) and expressed as the score for cartilage erosion, as described [34].

### Fluorescence microscopy

Kidneys and spleens of NZB/W mice were embedded in OCT compound (Sakura Finetek, Torrance, CA, USA) and snap-frozen in liquid nitrogen. Frozen sections were fixed in acetone and blocked with 10% normal donkey serum (Sigma Aldrich). Kidney sections were stained with a 1:200 dilution of anti-IgG-biotin (Sigma Aldrich), anti-IgM-biotin (Southern Biotech) and anti-C3-biotin (Bioss), followed by reaction with 1:200-dilution of streptavidin-cy3 (Invitrogen). Spleen sections were stained with anti-GL7-FITC, anti-CD4-APC, and anti-IgD-PE (all from BD Biosciences or eBioscience). Fluorescence images were acquired using a TCS SP5 confocal microscope (Leica). The area of GCs composed with GL7^+^ cells per image was calculated using ImageJ software (NIH, Bethesda, MD, USA).

### ELISA

Titers of anti-dsDNA IgG Ab were measured by ELISA, as described previously [35]. In brief, sera were diluted 1/10 000 to 1/50 000 in PBS and applied to immunosorbent plates (Nunc) precoated with 5 μg/ml poly-L-lysine and 5 μg/ml thymic DNA (Sigma Aldrich). The serum containing the highest titer of anti-dsDNA IgG was serially diluted and used as standard. The plates were incubated with anti-mouse IgG-biotin (Sigma Aldrich) and streptavidin-HRP (BD Biosciences). The concentration of albumin in urine was measured by quantitative ELISA using a mouse albumin ELISA kit (Bethyl), according to the manufacturer’s instructions. The concentration of serum B-cell activating factor of the TNF family (BAFF) was quantitated using a BAFF/BLys/TNFSF13B ELISA Kit (R&D Systems).

### FACS

Single-cell suspensions of spleen and LN cells were prepared as previously described [35]. Joint tissues from hind paws of KSTA mice were dissected free of soft tissue and bones, digested with 100 μg/ml Liberase (Roche) for 45 min at 37^o^C, and filtered through a 70-μm-pore cell strainer (SPL Life Sciences) to prepare single cell suspensions of synovial infiltrates. The single cell suspensions were stained with an appropriate combination of mAbs and analyzed by FACS. The mAbs used were: CD138-PE, B220-PerCP or -APC-cy7, FAS-PE, CD19-PerCP or -APC, CD21-FITC or -APC, CD43-APC, CD23-PE-cy7, CD8a-PE, CD25-APC-cy7, CXCR5-biotin, GL7-FITC, CD45.2-FITC, CD27-FITC, Ki-67-FITC, Gr-1-FITC, CD4-FITC or -APC-cy7, phospho-Syk-PE, CD11b-PE or -PerCP, PD-1-APC, CD44-APC-cy7, and F4/80-PerCP mAbs (all from BD Biosciences, eBioscience or Biolegend). Streptavidin-PerCP was purchased from BD Biosciences.

### Reverse transcription (RT) and quantitative PCR

Splenic CD19^+^ B cells from NZB/W mice were sorted by MACS, and lysed with Trizol reagent (Invitrogen) followed by standard methods of total RNA purification. The RNA was assayed by RT and quantitative PCR as described [36]. Levels of BAFFR transcripts were normalized to the level of β-actin transcripts. PCR primers used were 5ʹ-TCGACCCTCTGGTGAGAAAC-3ʹ and 5ʹ-CACGCTGCTTGTATGTCCAG-3ʹ for BAFFR and 5ʹ-GACGGCCAGGTCATCACTATTG-3ʹ and 5ʹ-AGGAAGGCTGGAAAAGAGCC-3ʹ for β-actin.

## Results

### SKI-O-592 is a potent and selective Syk kinase inhibitor

We have developed a novel pyrazolylpyrimidine compound named SKI-O-592 to selectively inhibit Syk. We also converted SKI-O-592 to SKI-O-703, a mesylate salt form with suitable physicochemical properties and ADME profile. SKI-O-703 is soluble in aqueous solution and can be used orally *in vivo*. The IUPAC name of SKI-O-703 is (S)-cyclopropyl(5-((4-(4-((4-hydroxyisoxazolidin-2-yl)methyl)-3-methyl-1H-pyrazol-1-yl)pyrimidin-2-yl)amino)-1-methyl-1H-indol-3-yl)methanone dimesylate, and its structure is shown in Supplementary Fig. S1A.

We first carried out kinase assays to determine how efficiently and selectively SKI-O-592 inhibits the kinase activity of Syk in comparison with R406, a well-known Syk inhibitor. SKI-O-592 and R406 inhibited the kinase activity of Syk with IC_50_ values of 6.2 nM and 56.5 nM, respectively, indicating that SKI-O-592 is approximately 9-fold more potent than R406 as a Syk kinase inhibitor (Table 1). SKI-O-592 was highly specific for Syk, since it had IC_50_ values against all other kinases we tested from 67- to 2753-fold higher than against Syk. Unlike SKI-O-592, R406 inhibited all these kinases except FGFR1 and AuroraB more efficiently than Syk. Thus SKI-O-592 is much superior to R406 as a Syk kinase inhibitor in terms of both potency and selectivity.

**Table 1:** SKl-0-592 specifically inhibits the kinase activity of Syk

Kinases	SKl-O-592		R406	
	IC_50_ (nM)	Ratio to Syk	IC_50_ (nM)	Ratio to Syk
Syk	6.2	1.0	56.5	1.0
Jak2	1859	302	1.3	<0.1
Jak3	5807	943	16.3	0.3
RET	412	67	10.7	0.2
KOR	687	111	18.8	0.3
FLT3	1783	289	0.5	<0.1
FGFR1	16 960	2753	88.9	1.6
FGFR2	>10 000	>1623	22.4	0.4
FGFR3	5662	919	32.2	0.6
Pyk2	709	115	24.3	0.4
AuroraB	>10 000	>1623	164.7	2.9

**Figure 1:**
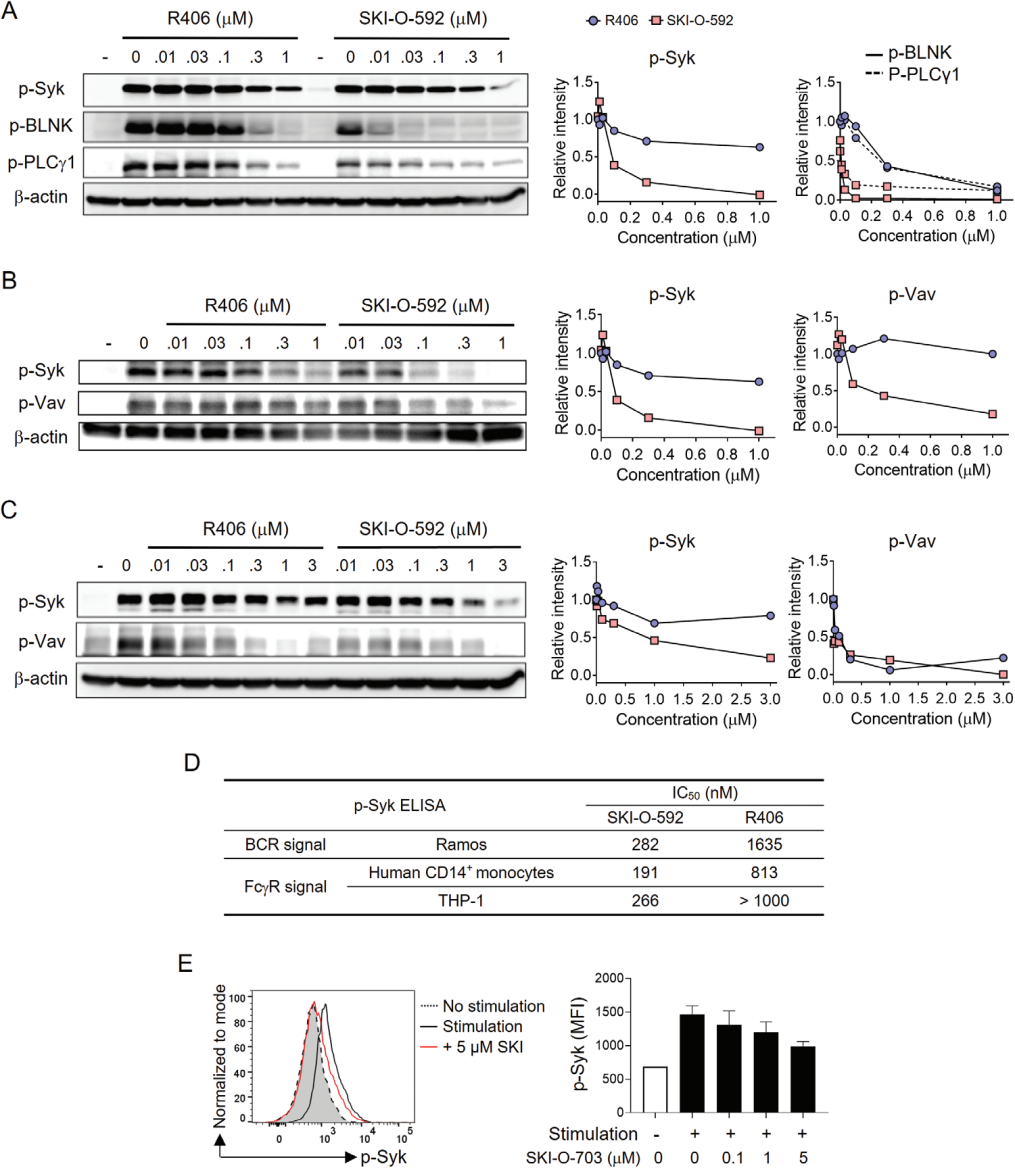
*in vitro* inhibitory effects of SKI-O-592 and -703 on Syk in B cells and monocytes. Ramos cells (**A**), human primary monocytes (**B**), and THP cells (**C**) were pretreated with inhibitors at the indicated concentrations and stimulated with anti-human IgM (A) and human IgG (B and C). The cells were then lysed and assayed by immunoblotting methods. The relative intensities of protein phosphorylation levels were quantitated with reference to each lane of β-actin control bands. (**D**) Ramos cells, human primary monocytes, and THP cells were treated as in A–C for 24 h and levels of phosphorylated Syk (p-Syk) were measured by ELISA. (**E**) Mouse primary B cells were stimulated with anti-IgM mAb for 60 min in the presence or absence of SKI-O-703 and assayed for p-Syk by FACS. A representative FACS profile and data on the dose-response of mean fluorescence intensity (MFI) are shown. The data are representative of two independent experiments.

Phosphorylation of tyrosine residues in Syk is required for its activity. To determine whether SKI-O-592 inhibits the phosphorylation of Syk that occurs upon crosslinking BCR and FcγR, we cultured Ramos human B cells and human monocytes (primary CD14^+^ monocytes and THP-1 cells) with anti-IgM and IgG, respectively, and measured phosphorylated Syk by immunoblotting. SKI-O-592 treatment inhibited phosphorylation of tyrosine residues 525 and 526 of Syk, and its downstream molecules (BLNK, PLCγ1, and Vav1), in all three cell lines in a dose-dependent manner, and had a more potent effect than R406 (Fig. 1A–C). According to measurements of phosphorylated Syk by ELISA, the IC_50_ value of SKI-O-592 was 3.7- to 5.8-times lower than that of R406 in all three cell lines (Fig. 1D). Thus, these results confirm that SKI-O-592 inhibits the phosphorylation and subsequent kinase activity of Syk in B cells and monocytes more efficiently than R406. Consistent with this, treatment with SKI-O-703 (a mesylate salt of SKI-O-592) reduced the phosphorylation of Syk in response to BCR and CD40 stimulation in mouse primary B cells (Fig. 1E).

Zap70 kinase transduces T cell receptor (TCR)-mediated signals in T cells in a manner similar to that of Syk in B cells. We therefore tested whether SKI-O-592 inhibited Zap70 in T cells. SKI-O-592 at 0.3–10 μM failed to affect the phosphorylation of Zap70 and its downstream target PLCγ1 in TCR-stimulated T cells, whereas these phosphorylation events were inhibited by ≥ 1.1 μM R406 (Supplementary Fig. S1B). Thus, SKI-O-592 appears to be ineffective in inhibiting TCR-mediated signaling in T cells.

### SKI-O-592 and -703 selectively inhibit BCR-mediated survival, proliferation, and differentiation of B cells

**Figure 2:**
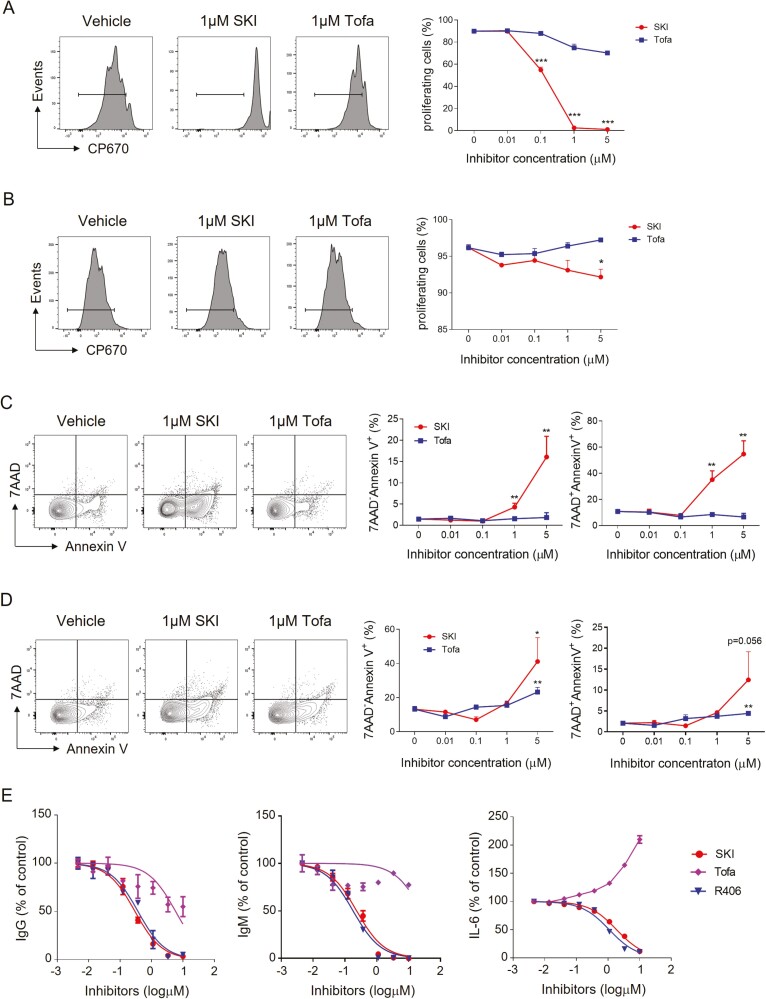
*in vitro* effects of SKI-O -703 on the proliferation, survival, and differentiation of B cells. Mouse primary B cells were labeled with CP670, and stimulated with either anti-IgM mAb, CD40L and IL-4 (**A**) or LPS (**B**) for 72 h in the presence or absence of SKI-O-703 and tofacitinib at the indicated concentrations; they were then assayed by FACS. (**C** and **D**) Mouse primary B cells were cultured as in A and B for 24 h, stained with Annexin V and 7-AAD, and assayed by FACS. Representative FACS profiles and graphs displaying mean Annexin V-positivity are shown. (**E**) Human primary B cells were stimulated with CpG ODN plus IL-2 (to detect IgM and IgG) or CpG ODN 2006 plus IFN-α (to detect IL-6) in the presence or absence of inhibitors. The culture supernatants were assayed by ELISA. Graphs are expressed as % positivity relative to the controls (without inhibitor). The data are representative of 2–3 independent experiments. *P < 0.05, **P < 0.01, and ***P < 0.001, compared with vehicle control by two-tailed unpaired Students *t*-tests.

Given that Syk-mediated signaling is critical for the survival, proliferation, and differentiation of B cells, we asked whether SKI-O-703 interfered with these processes in primary B cells. We stimulated mouse primary B cells with either anti-IgM mAb or LPS in the presence or the absence of SKI-O-703 and examined these phosphorylation events. In the CP670-dilution assay to detect dividing cells, 0.1–5 μM SKI-O-703 inhibited BCR crosslinking-induced proliferation in a concentration-dependent manner (Fig. 2A). LPS-induced proliferation was less susceptible to SKI-O-703, since at least 5 μM SKI-O-703 was required to significantly inhibit proliferation (Fig. 2B).

When early apoptotic (AnnexinV^+^7-AAD^−^) and late apoptotic (AnnexinV^+^7-AAD^+^) cells were detected by FACS, SKI-O-703 treatment increased the proportions of early and late apoptotic cells and this cytotoxic effect was more potent when the B cells were activated by anti-IgM mAb than by LPS (Fig. 2C and D). This observation demonstrates that SKI-O-703 selectively inhibits survival and proliferation in response to the BCR-Syk signaling axis and supports the idea that Syk acts proximal to BCR rather than prior to TLR4. To confirm that this cytotoxicity is associated with selective inhibition of Syk activity, we tested the effect of SKI-O-703 on the survival of TCR-engaged primary T cells and found that up to 5 μM SKI-O-703 failed to increase the proportions of early and late apoptotic cells (Supplementary Fig. S1C).

We next tested whether SKI-O-592 was able to inhibit the differentiation of B cells into Ab- and IL-6-secreting cells. For this purpose, we cultured human primary B cells stimulated with a TLR9 agonist plus cytokines (IFN-α or IL-2) in the presence or absence of inhibitors and performed ELISAs on culture supernatants. SKI-O-592 inhibited the production of IgM, IgG, and IL-6, with an efficacy comparable to that of R406 (Fig. 2E). SKI-O-592 also inhibited IL-2 production by activated T cells, but with potency about 10-fold lower than that of R406 (Supplementary Fig. S1D). These results demonstrate that SKI-O-592 efficiently blocks the differentiation of B cells into Ab- and proinflammatory cytokine-secreting cells.

We also tested the effect of the Jak-1 and Jak-3 inhibitor, tofacitinib (CP-690550), and found that tofacitinib up to 1 μM did not significantly inhibit the proliferation and death of primary B and T cells (Fig. 2A–D, Supplementary Fig. S1C). It had a minimal effect on Ab production and even increased IL-6 production (Fig. 2E). This result suggests that SKI-O-703 might be more effective than tofacitinib in reducing B cell activation-mediated pathology.

### Orally administered SKI-O-703 reduces lupus nephritis-like manifestations in NZB/W mice

NZB/W female mice have been used as models of the multifactorial complexity of SLE. Disease development in this model is largely dependent on GC-driven Ab responses and subsequent type III hypersensitivity, which is most prominent in kidney tissues [37]. We used the NZB/W model to see whether SKI-O-703 inhibited the onset and progression of lupus nephritis by suppressing the activation of autoreactive B cells, and autoAb production. NZB/W female mice at 18 weeks of age contained about 27- to 81-fold higher titers of anti-dsDNA IgG Ab than normal C57BL/6 mice, without any overt pathologic manifestations (data not shown). Female NZB/W mice at this age, namely at the autoimmunity-established preclinical phase, were assigned to four groups, and the groups were administered orally with 42 mpk of SKI-O-703, 84 mpk of SKI-O-703, a control drug, or vehicle, respectively. Thrity mpk of tofacitinib was used as a control drug, according to a previous study [38]. The drugs and vehicle were given once daily up to 34 weeks of age.

**Figure 3:**
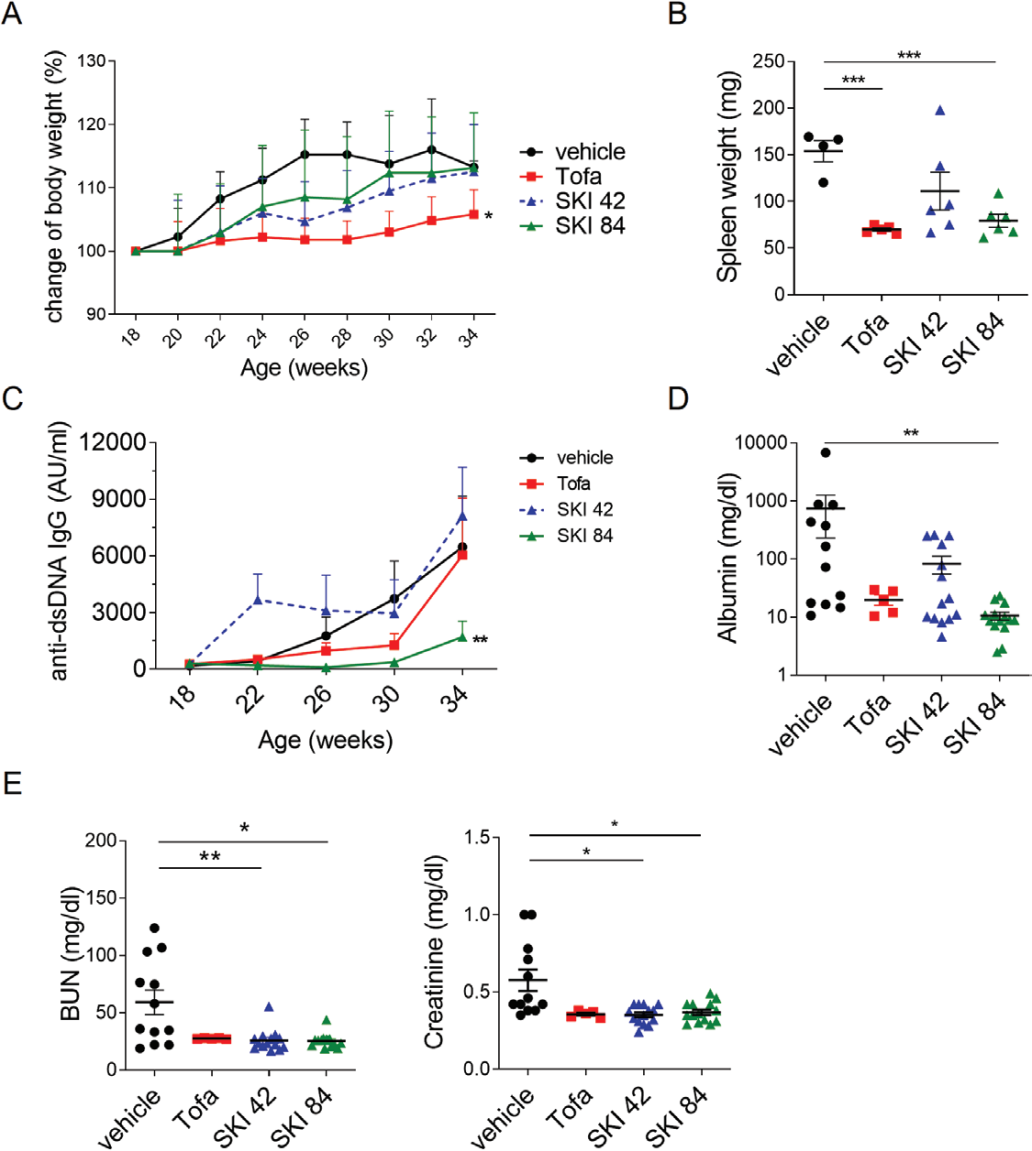
*in vivo* effect of oral SKI-O-703 on lupus-like signs in NZB/W mice. Female NZB/W F1 mice were administrated orally with 42 mpk of SKI-O-703 (SKI 42), 84 mpk of SKI-O-703 (SKI 84), or tofacitinib (Tofa) from 18 to 34 weeks of age. (**A**) Body weights were measured every 2 weeks and are displayed as mean percent change of body weight, with SEM. (**B**) Spleen weights measured at the end of the experiment. (**C**) Serum was collected every 4 weeks and assayed by ELISA to measure titers of anti-dsDNA IgG. Mean titers are shown. AU, arbitrary unit. (**D**) Urine collected at 34 weeks of age and urinary albumin was assayed by ELISA. (**E**) Concentrations of blood urine nitrogen (BUN) and creatinine at 34 weeks of age. Graphs display means ± SEMs, and symbols represent values of individual mice (B, D, and E). *P < 0.05, **P < 0.01, and ***P < 0.001, compared with the vehicle control group by two-way ANOVA (A and C) and two-tailed unpaired Students *t*-test (B, D, and E).

Splenomegaly is a hallmark of SLE. SKI-O-703 treatment at high dose (84 mpk), but not low dose (42 mpk), significantly reduced spleen weights without significantly affecting body weights, indicating that it reduced splenomegaly without causing wasting (Fig. 3A and B). By contrast, exposure to tofacitinib reduced both body weights and spleen weights.

During the period of treatment (18–34 weeks of age), the serum level of anti-dsDNA IgG Ab gradually rose in the vehicle control group, and this effect was dramatically attenuated by 84 mpk of SKI-O-703 (*P* < 0.01 by two-way ANOVA test) (Fig. 3C). Treatment with 42 mpk of SKI-O-703 did not have the same effect, and even temporarily elevated the Ab titer. There was a trend towards reduced Ab titer at 26 and 30 weeks of age in the tofacitinib-treated group, but it did not reach statistical significance.

Levels of urinary protein, blood urea nitrogen (BUN), and blood creatinine are indicators of lupus nephritis and renal dysfunction. We measured these markers at 34 weeks of age. The mean concentration of urinary albumin was 742.9 mg/dl in the vehicle control group, and this was markedly reduced in the group dosed with 84 mpk SKI-O-703, but not in the 42 mpk group (Fig. 3D). The concentrations of BUN and creatinine were significantly lowered in both the high-dose and low-dose-treatment groups. Taken together these findings demonstrate that SKI-O-703 can inhibit splenomegaly, autoantibody production, and renal dysfunction in NZB/W mice.

### Oral administration of NZB/W mice with SKI-O-703 attenuates the histopathological manifestations of glomerulonephritis

**Figure 4:**
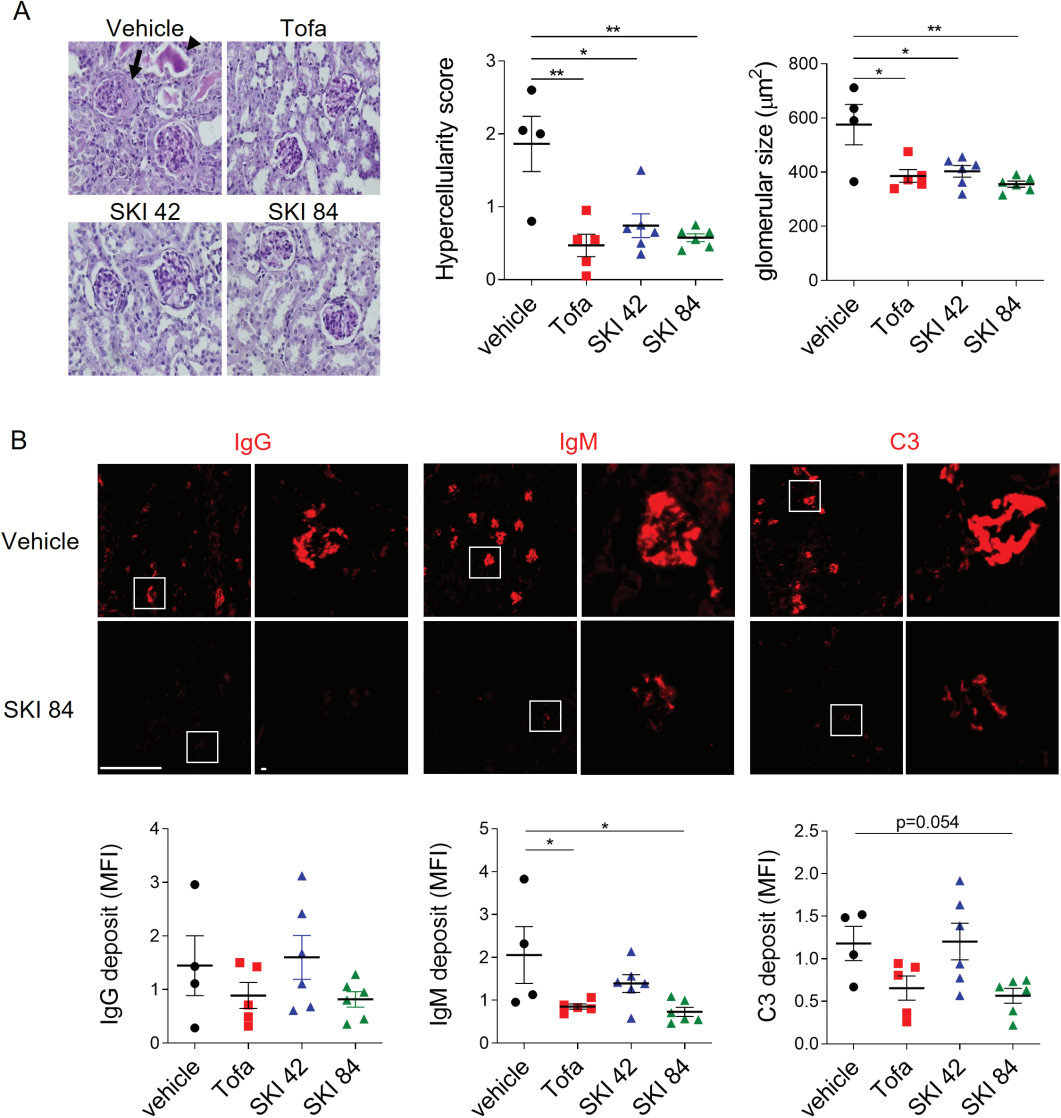
histopathological alteration of kidney tissues by SKI-O-703 in NZB/W mice. Female NZB/W F1 mice were administrated orally with 42 mpk SKI-O-703 (SKI 42), 84 mpk SKI-O-703 (SKI 84), or tofacitinib (Tofa) from 18 to 34 weeks of age, and kidneys were examined at 34 weeks by histopathological methods. (**A**) Paraffin sections were stained with PAS and hematoxylin. An eosinophilic protein cast and crescent are indicated by the arrowhead and arrow, respectively. The images are representative of each group. Graphs show means ± SEMs with symbols representing values of individual mice. (**B**) Cryosections were stained with anti-IgG, anti-IgM, and anti-C3 Abs and observed by fluorescence confocal microscopy. Boxes in the left images are magnified in the right images. Representative images with mean fluorescence intensities are shown. Bar scale, 512 μm. *P < 0.05 and **P < 0.01 by two-tailed unpaired Students *t*-test.

The histopathologic manifestations of lupus nephritis include glomerular hypercellularity, glomerular enlargement, and the appearance of eosinophilic protein casts and crescents. All these manifestations were clearly evident in the vehicle control group and significantly improved in all three drug-treated groups, as eosinophilic protein casts and crescents were absent and glomerular hypercellularity and size were significantly reduced (Fig. 4A).

Since immune complexes along with complement factors are deposited into glomeruli and then induce inflammatory responses, we examined whether SKI-O-703 treatment blocked these pathologic processes. Once again, whereas the glomeruli of the kidneys from the vehicle control mice were highly stained with fluorescence-labeled mAbs to IgG, IgM, and C3, the intensities of IgM and C3 staining were significantly reduced in the high dose, but not low dose, SKI-O-703 group (Fig. 4B).

### Oral administration of SKI-O-703 to NZB/W mice reduces the GC- and BAFF-signaling involved in humoral immune responses

**Figure 5:**
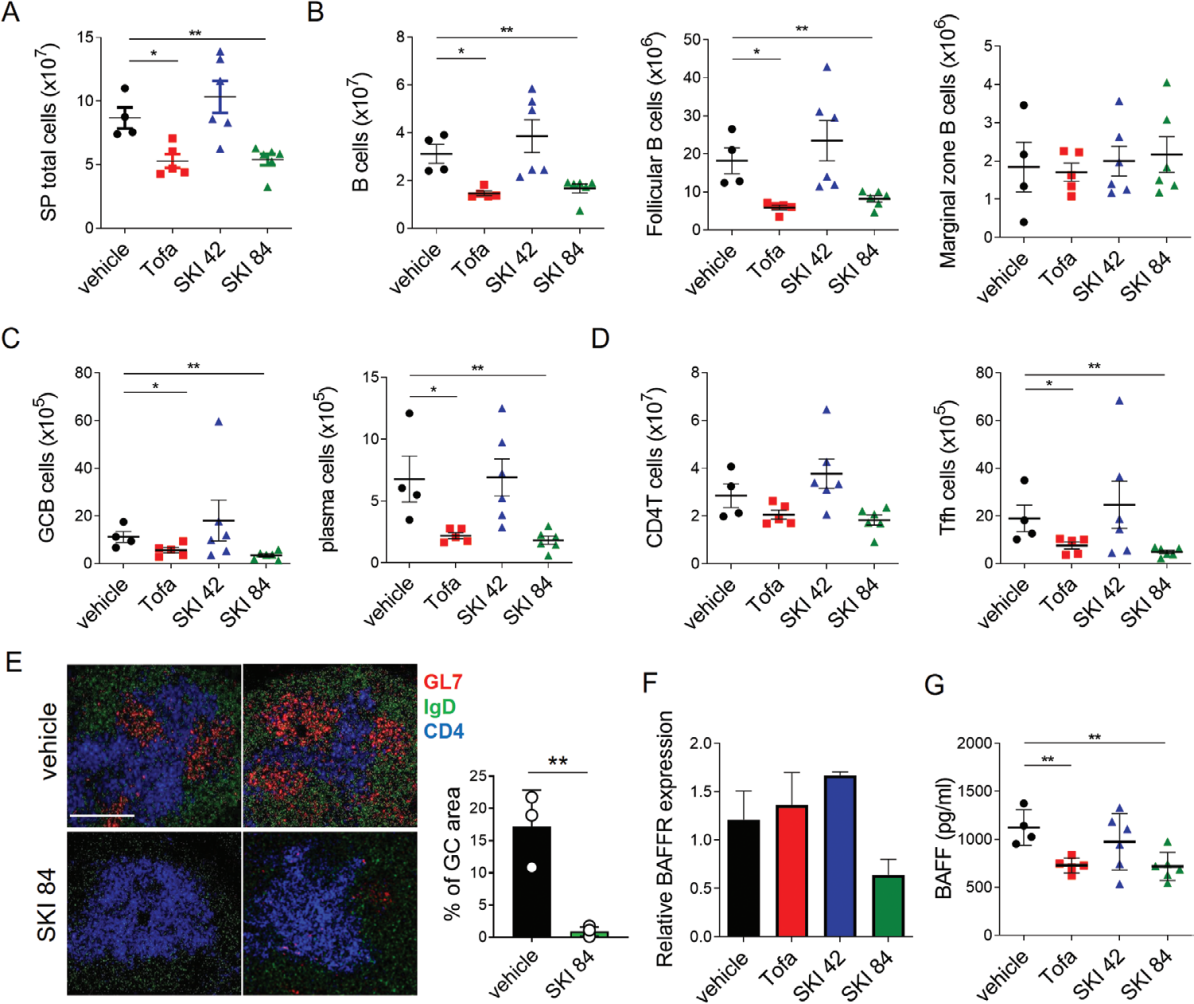
reduced numbers of spleen cells in SKI-O-703-treated NZB/W mice. Female NZB/W F1 mice were administrated orally with 42 mpk SKI-O-703 (SKI 42), 84 mpk SKI-O-703 (SKI 84), or tofacitinib (Tofa) from 18 to 34 weeks of age. Spleens and sera were collected at 34 weeks of age. (**A**–**D**) Spleens were assayed by FACS. The gating strategy is displayed in Supplementary Fig. S2. (**E**) Spleens were cryosectioned, stained with Abs to GL-7, IgD, and CD4, and analyzed by confocal microscopy. Two representative images per group are shown. Bar scale, 80 μm. Percentage of GC area (area of GL7^+^/area of total area) from 3 to 4 images/group are displayed as mean ± SD. (**F**) Splenic B cells were assayed by quantitative RT-PCR. Levels of BAFFR mRNA were normalized to the level of β-actin mRNA. (**G**) Concentrations of BAFF measured by ELISA in sera. The data are representative of at least two independent experiments. Graphs show means + SEMs, and symbols represent values of individual mice. *P < 0.05 and **P < 0.01 by two-tailed unpaired Students *t*-test.

To address the cellular and molecular mechanisms by which SKI-O-703 attenuates lupus nephritis in NZB/W mice, we collected spleens, BM, and sera at 34 weeks of age, and examined them by FACS and biochemical methods. Consistent with the reduced splenomegaly, total spleen cells were significantly less numerous in the high dose SKI-O-703 group (Fig. 5A). This was mainly due to a reduction in the B cell population, because the number of T cells was not significantly altered (Fig. 5B and D). All subsets of B cells, follicular, GC, and plasma cells, but not marginal zone cells, were significantly less numerous than those of vehicle controls (Fig. 5C and Supplementary Fig. S2). In the plasma cells, the ratio of Ki-67^+^ dividing cells to Ki-67^-^ non-dividing cells was reduced, indicative of selective deletion of short-lived plasma cells (Supplementary Fig. S3). Despite the unaltered number of CD4^+^ T cells, CXCR5^+^PD-1^+^ Tfh cells were reduced in number (Fig. 5D). Moreover, fluorescence microscopy revealed fewer GL7^+^ GC B cells and IgD^+^ follicular B cells in the spleens of SKI-O-703-treated mice than in the vehicle controls (Fig. 5E).

To see whether long-term treatment with SKI-O-703 affected BM hematopoiesis, we determined the cellular composition of BM cells and found that the proportions and numbers of the various BM cell populations did not differ between the groups. In particular, all B lineage-committed cells, such as prepro-, pro-, pre- and immature B cells, normally populated in the BM of SKI-O-703-treated mice (Supplementary Fig. S4). Therefore, SKI-O-703 appeared not to interfere with BM hematopoiesis, and the reduction in B cells did not result from defective B lymphopoiesis in the BM.

The cytokine BAFF is critical for the survival and differentiation of B cells and plays a role in the development of SLE [39]. We found, interestingly, that B cells exposed to high dose SKI-O-703 expressed the BAFF receptor at a reduced level (Fig. 5F), and the level of serum BAFF was also reduced (Fig. 5G). Therefore, the effect of SKI-O-703 on the suppression of lupus nephritis was mirrored by the reduced BAFF-BAFF receptor system.

### SKI-O-703 inhibits KSTA by reducing recruitment of neutrophils and macrophages into synovial tissue

**Figure 6:**
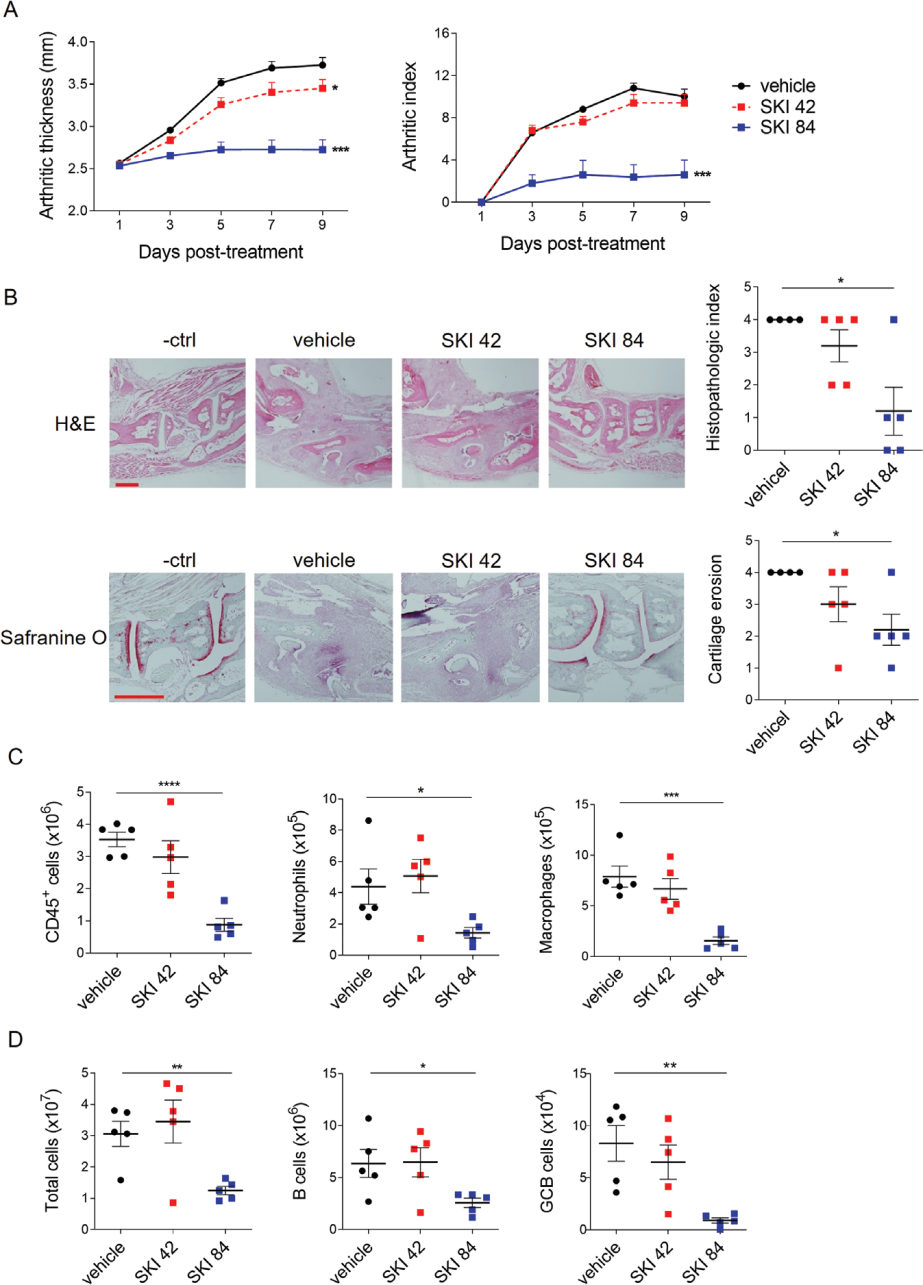
SKI-O-703 attenuates KSTA. BALB/c mice were infused with K/BxN serum and orally dosed with 42 mpk SKI-O-703 (SKI 42) or 84 mpk SKI-O-703 (SKI 84) twice a day for 9 days. (**A**) Ankle thickness and arthritic indexes are displayed as means + SEMs. (**B**) On Day 9, hind paws were examined by histopathologic methods after staining with H&E and Safranine O. Representative images and graphs displaying means ± SEMs with symbols representing each individual are shown. (**C**) Spleen cells and joint-draining LN cells were assayed by FACS. Graphs display means ± SEMs, with symbols representing values of individual mice. The data are representative of four independent experiments. *P < 0.05, **P < 0.01, and ***P < 0.001, compared with the vehicle control group by two-way ANOVA (A) and two-tailed unpaired Student’s *t*-test (B-**D**).

AutoAb-mediated inflammatory responses are evident in human RA as well as SLE, and this effect is well illustrated in the KSTA mouse model. To distinguish the effect of SKI-O-703 on innate inflammatory cells from that on B cells, we orally dosed normal BALB/c mice with SKI-O-703 during the progression of KSTA. We found that both ankle thickness and arthritic index were dramatically reduced to the basal level in 84 mpk SKI-O-703-treated mice and the reduction was less pronounced at the lower dose (Fig. 6A). Both histopathologic index and cartilage erosion score were significantly reduced only in the high dose mice (Fig. 6B).

AutoAbs deposited in the synovial tissue can elicit innate inflammatory responses by recruiting and activating neutrophils and macrophages. Indeed, these cells are the main agents of KSTA [21, 40]. As expected, SKI-O-703 treatment at 84 mpk, but not 42 mpk, significantly reduced numbers of whole immune cells (CD45^+^) infiltrated into synovial tissue. Neutrophils and macrophages were the main cell types whose numbers were strongly affected (Fig. 6C). Minor cell types, such as B cells, CD4^+^ T cells, and plasma cells, were also significantly less numerous in the high dose mice (data not shown). Thus, these results demonstrate that SKI-O-703 can suppress macrophage- and neutrophil-mediated inflammatory activation at sites of autoAb deposition.

Interestingly, despite the acute synovitis induced by arthritogenic serum transfer, the joint-draining LNs from the mice receiving K/BxN serum exhibited lymphadenopathy on Day 9 post-infusion. This pathologic manifestation disappeared upon high dose SKI-O-703 treatment, which also resulted in significantly reduced numbers of total cells, B cells and GC B cells (Fig. 6D), indicating that SKI-O-703 is effective in preventing episodes of innate inflammation in local tissue from developing into adaptive immune activation in draining LNs.

### Combined treatment with TNF blockade and SKI-O-703 suppresses KSTA

**Figure 7:**
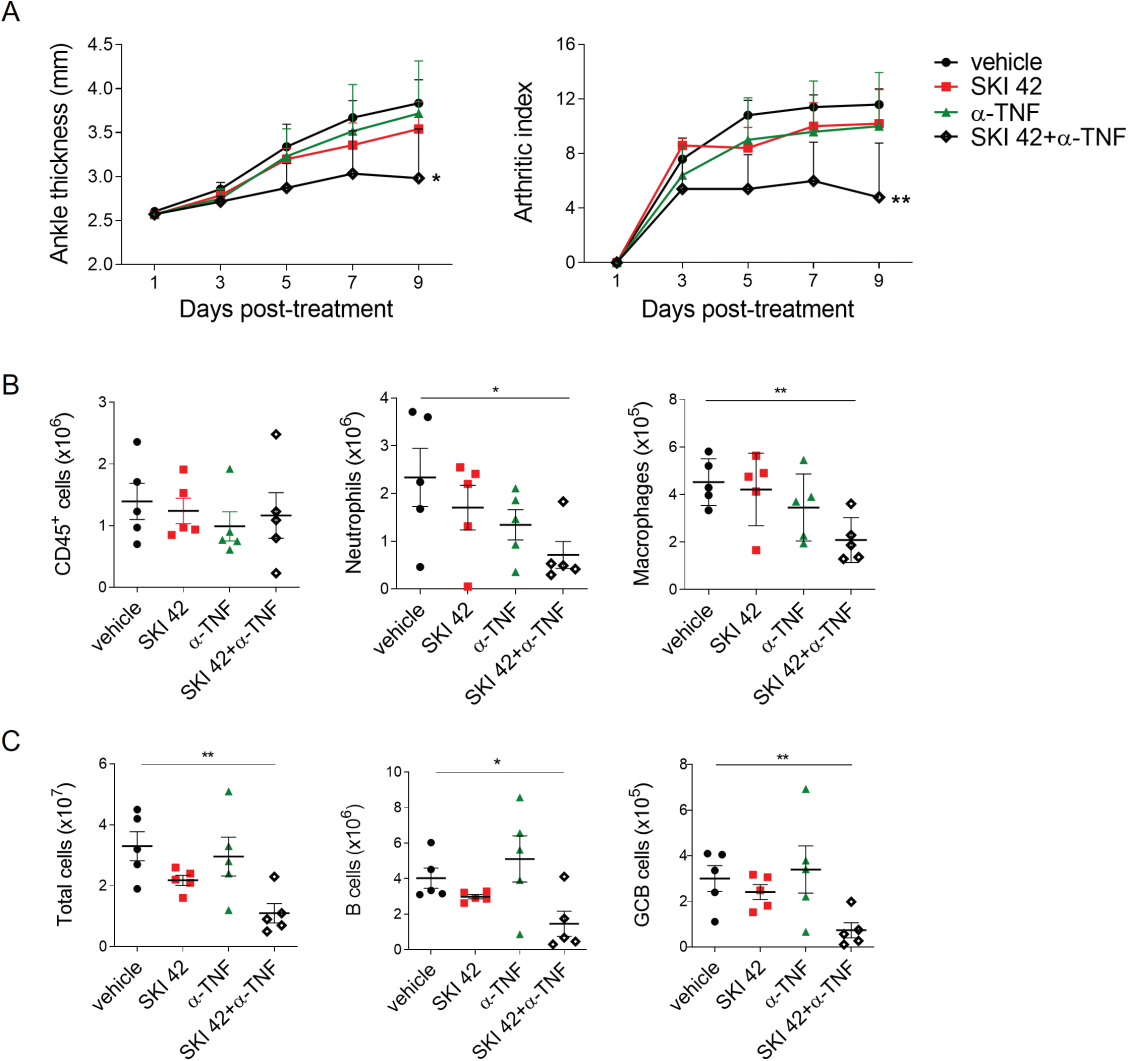
effects of SKI-O-703 combined with TNF blockade on KSTA. BALB/c mice were infused with K/BxN serum and treated with 42 mpk SKI-O-703 (SKI 42) alone, etanercept (a-TNF) alone, or both, for 9 days. (**A**) Ankle thickness and arthritic indexes on a given day are shown as means + SEM. (**B** and **C**) Spleen and dLN cells were analyzed by FACS. The data are representative of four independent experiments. *P < 0.05, **P < 0.01, and ***P < 0.001, compared with the vehicle control group by two-way ANOVA (A) and two-tailed unpaired Student’s *t*-test (B and C).

TNF is a proinflammatory cytokine that plays a critical role in the progression of RA and KSTA. Because of this, anti-TNF treatments have been used in many inflammatory diseases. Nevertheless, a substantial proportion of RA patients are refractory to anti-TNF therapy [41] and the effects of single treatments of TNF blockade in animal models of RA are subtle or uncertain [42, 43]. Consistent with this, we found that treatment of mice with up to 400 μg/mouse of the TNF blocker eternacept (a soluble TNF receptor II-human IgG1 Fc fusion protein) did not significantly inhibit progression of KSTA, as was true for suboptimal doses (42 mpk) of SKI-O-703. However, when we combined the two treatments they had a synergistic effect and significantly suppressed disease progression, as judged by the reductions in ankle thickness, arthritic index, and infiltration of innate inflammatory cell into joints and draining LNs (Fig. 7). Thus, SKI-O-703 at a suboptimal dose is a candidate drug for use in combination with anti-TNF treatments.

## Discussion

In the present study, we have presented *in vitro* and *in vivo* evidence that a novel selective Syk inhibitor, SKI-O-703, that we have developed can reduce SLE- and RA-like symptoms in animal disease models. The underlying mechanism involves blocking BCR- and FcR-proximal signals that are essential for activation of B cells and innate inflammatory cells, respectively. Therefore, SKI-O-703 appears to be a promising drug candidate for treatment of Ab-mediated inflammatory diseases, targeting both Ab-producing and Ab-sensing cells.

IgG class-switched affinity-matured autoAbs are the cause of lupus and are mainly produced as a result of GC reactions. The coordinated events taking place in the GC are tightly controlled by a variety of mechanisms including Syk signals. For example, the Syk-Mcl1 pathway enables GC-like B cells to survive, and inhibition of that pathway induces apoptosis of those cells [14]. A cell-autonomous function of Syk in GC B cells is required for their differentiation into plasma cells, and Syk degradation interferes with plasma cell formation in GCs [13]. We found above that the reduction of plasma cell numbers due to SKI-O-703 was associated with a reduction in GC B cells. This suggests that Syk inhibition by SKI-O-703 suppresses the differentiation of follicular B cells into plasma cells via the GC pathway, thereby leading to reduced production of IgG autoAb. The idea that the “follicular B-GC B-plasma cell” axis is the target of SKI-O-703 is supported by our observation that SKI-O-703-treated mice had reduced numbers of Tfh cells, a population responsible for positive selection of GC B cells in the GC.

Although the impact of SKI-O-703 on follicular B cells was obvious, we found, interestingly, that it did not alter the size of the marginal zone B cell population. Marginal zone B cells constitute a subset of mature B cells specifically dwelling in marginal zones of the spleen. In addition to phenotypic differences, they have functional features distinct from follicular B cells, as they readily mount rapid Ab responses to blood-borne antigens via a pathway that does not require the GC reaction [44]. They also maintain their numbers in a manner more dependent on TLR signals than is the case for follicular B cells [45, 46]. These characteristics may explain why marginal zone B cells were resistant to SKI-O-703 treatment. This interpretation is also consistent with our finding that SKI-O-703 did not significantly alter TLR4-induced proliferation of B cells. Maintaining the marginal zone B cell population upon SKI-O-703 treatment while suppressing the immune competence of follicular B cells might be beneficial in protecting the host from blood-borne infectious agents. Given that many immunosuppressive drugs have unwanted complications, such as enhancing susceptibility to infectious agents [47], our data suggest that SKI-O-703 may provide a superior therapeutic strategy by selectively targeting the follicular to GC B cell pathway without reducing marginal zone B cell function.

Importantly, we have demonstrated that SKI-O-703 does not alter the early development of B-lineage cells or of other hematopoietic cells, as judged by the normal proportions of all early progenitors, myeloid- and B lymphoid-lineage cells in the BM of mice treated with SKI-O-703 for a long period (16 weeks). This finding provides robust evidence that SKI-O-703 does not perturb hematopoiesis, as long-term treatment with immunosuppressants frequently does [48]. Furthermore, given that lymphopenia is prone to precipitate homeostatic proliferation-driven autoimmune states [49, 50], SKI-O-703 may not amplify autoimmunity via such a lymphopenia-associated compensatory mechanism.

It is noteworthy that, although the number of plasma cells was decreased upon SKI-O-703 treatment, the ratio of non-dividing to dividing plasma cells increased. This result implies that non-dividing long-lived plasma cells, unlike dividing short-lived plasma cells, are refractory to the cytotoxic effect of SKI-O-703. This result is not surprising, since a hallmark of long-lived plasma cells is resistance to most cytostatic drugs (e.g. cyclophosphamide) [51, 52]. Because one aspect of the action of vaccines is the preservation of long-lived plasma cells targeting pathogenic microbes, this lack of effect of SKI-O-703 on long-lived plasma cells would be beneficial in maintaining immune competence against microbial infection.

Since Syk is known to be relatively inert in T cells, we did not expect SKI-O-703 to inhibit T cell activation. Indeed, we found that T cells were refractory to the cytotoxic effects of SKI-O-703, unlike B cells. Moreover, T cells were less susceptible to SKI-O-703 than to R406 and tofacitinib in terms of inhibition of IL-2 production. Given that the regulatory T cells that are crucial for maintaining self-tolerance require IL-2 for their survival and function [53, 54], this property of SKI-O-703 should contribute to the maintenance of regulatory T cell-mediated self-tolerance.

Because of the complexity of inflammatory processes, optimal therapeutic efficacy would be achieved by inhibiting more than one mechanism. Indeed, we found that concurrent treatment by TNF blockade and SKI-O-703 at a suboptimal dose was more effective than the separate treatments in inhibiting the synovitis of KSTA. This finding suggests that use of SKI-O-703 in combination with TNF blockades could be attractive for treating RA. Such a combined strategy might help cope with various anti-TNF therapy-associated complications, such as the refractoriness of certain groups of patients, elevated infection rates, and high treatment costs.

In summary, our data provide evidence that SKI-O-703 is a highly selective and potent Syk inhibitor *in vitro* and suppresses Ab-mediated inflammatory diseases *in vivo*. It also acts synergistically with anti-TNF therapy. Thus, we propose that it is a promising candidate for the treatment of Ab-mediated inflammatory diseases.

## Supplementary Material

uxac096_suppl_Supplementary_Data

## Data Availability

The data that support the findings of this study are available from the corresponding author, [J.Y], upon reasonable request.

## Abbreviations

7-AAD: 7-aminoactinomycin D
Ab: antibody
APC: allophycocyanin
autoAb: autoantibody
BAFF: B-cell activating factor of the TNF family
BCR: B cell receptor
BM: bone marrow
dsDNA: double-stranded DNA
FITC: fluorescein isothiocyanate
GC: germinal center
H&E: hematoxylin and eosin
Ig: immunoglobulin
ITAM: immunoreceptor tyrosine-based activation motif
KSTA: K/BxN serum-transferred arthritis
LN: lymph node
LPS: lipopolysaccharide
mAb: monoclonal antibody
mpk: mg per kg body weight
NOD: non-obese diabetic
PAS: periodic acid-Schiff
PE: phycoerythrin
PerCP: peridinin chlorophyll
RA: rheumatoid arthritis
SLE: systemic lupus erythematosus
Syk: spleen tyrosine kinase
TCR: T cell receptor
Tfh: follicular helper T
TLR: Toll-like receptor
TNF: tumour necrosis factor.

## Permission to reproduce

Not Applicable. All content is the original work of the authors.
