# Supplementary material for: A novel selective spleen tyrosine kinase inhibitor SKI-O-703 (cevidoplenib) ameliorates lupus nephritis and serum-induced arthritis in murine models
Source: Clin Exp Immunol. 2022 Nov 8;211(1):31–45. doi: 10.1093/cei/uxac096 (PMC9993459; doi:10.1093/cei/uxac096)

Supplementary Figure 1. SKI-O-592 and -703 spare TCR-mediated signaling in T cells

(A) The structure of SKI-O-703. (B-D) Human peripheral T cells sorted from human peripheral blood mononuclear cells (B and D) and mouse primary T cells sorted from spleen (C) were stimulated with 5 μg/ml anti-CD3 and 2 μg/ml anti-CD28 mAbs in the presence or absence of inhibitors as indicated. After one hour (B) and 24 hours (C and D) of culture, cells were assayed by immunoblotting (B), FACS (C), and ELISA (D). SKI-O-592 did not inhibit phosphorylation of Zap70 and its downstream target PLCγ1. It's effect on the suppression of IL-2 production was 10- and 25-fold lower than those of R406 and tofacitinib, respectively, in terms of IC_{50}. SKI-O-703 at the tested concentrations did not promote apoptotic cell death of T cells.

Supplementary Figure 2. Gating strategy for FACS of spleen cells

Erythrocyte-depleted mouse spleen cells were assayed by FACS. Whole live cells were gated as shown: plasma cells ($CD19^{lo/-}$ $CD138^{hi}$), GC B cells ($CD19^{+}$ $CD138^{-}$ $GL\text{-}7^{hi}$ FAS^{hi}), follicular B cells ($CD19^{+}$ $CD43^{-}$ $CD23^{hi}$ $CD21^{-}$), marginal zone B cells ($CD19^{+}$ $CD43^{-}$ $CD21^{hi}$ $CD23^{lo}$), and Tfh cells ($CD4^{+}$ $CXCR5^{+}$ $PD\text{-}1^{hi}$).

Supplementary Figure 3. Differential susceptibility of short-lived and long-lived plasma cells to SKI-O-703

When spleen cells from NZB/W mice were assayed by FACS as in Fig. 5, they were stained intracellularly with anti-Ki-67 mAb to distinguish $Ki\text{-}67^{+}$ dividing cells from $Ki\text{-}67^{-}$ non-dividing cells among the $CD138^{hi}$ population. The graphs demonstrate that $Ki\text{-}67^{+}$ dividing (namely short-lived) plasma cells were more susceptible to SKI-O-703 than $Ki\text{-}67^{-}$ non-dividing plasma cells.

Supplementary Figure 4. Normal hematopoiesis in the BM of mice treated with SKI-O-703

Female NZB/W F1 mice were administrated orally with 12, 42 or 84 mpk of SKI-O-703 (labelled as SKI 21, SKI 42, SKI 84, respectively) as in Figure 3. BM was harvested from femurs at the end of the experiment. A, FACS gating strategy. B, Cell numbers. Symbols represent individual mice.

Supplementary Figure 5. Gating strategy for FACS of synovial cells

Single cell suspensions prepared from synovial tissues were assayed by FACS. Whole live cells were gated as shown: neutrophils ($Gr\text{-}1^{hi}$ $CD11b^{hi}$) and macrophages ($Gr\text{-}1^{lo}$ $F4/80^{hi}$).

Supplementary Figure 1

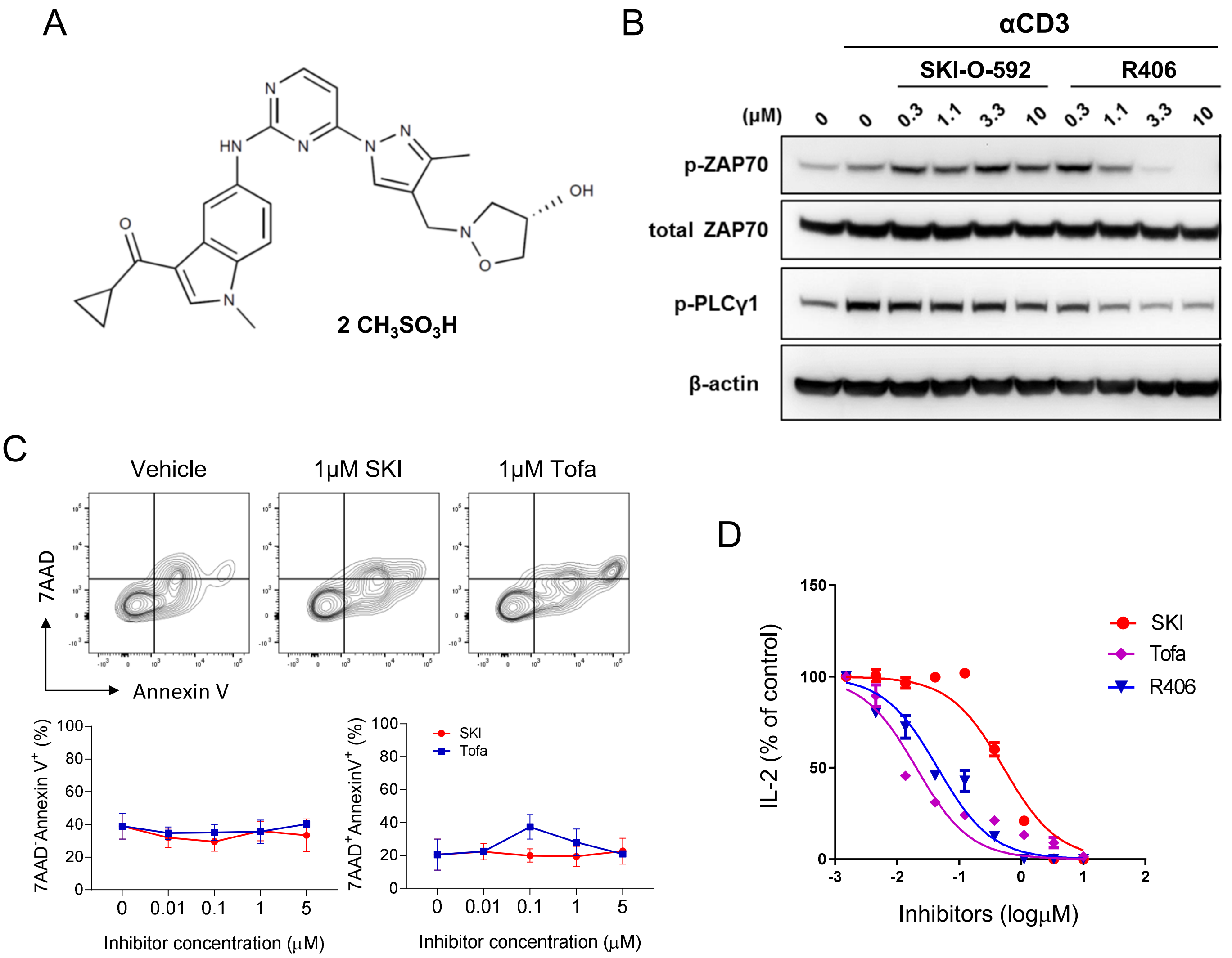

Supplementary Figure 2

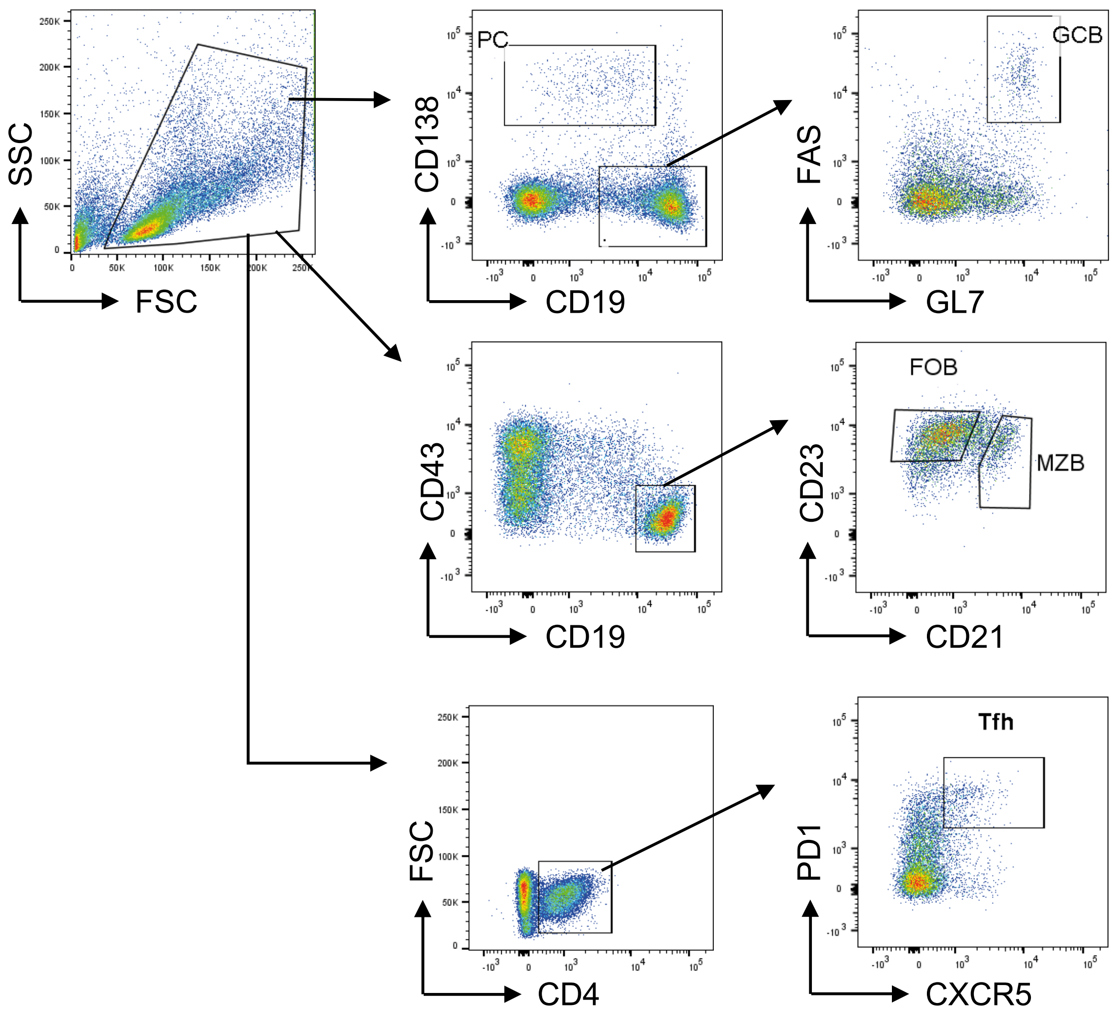

Supplementary Figure 3

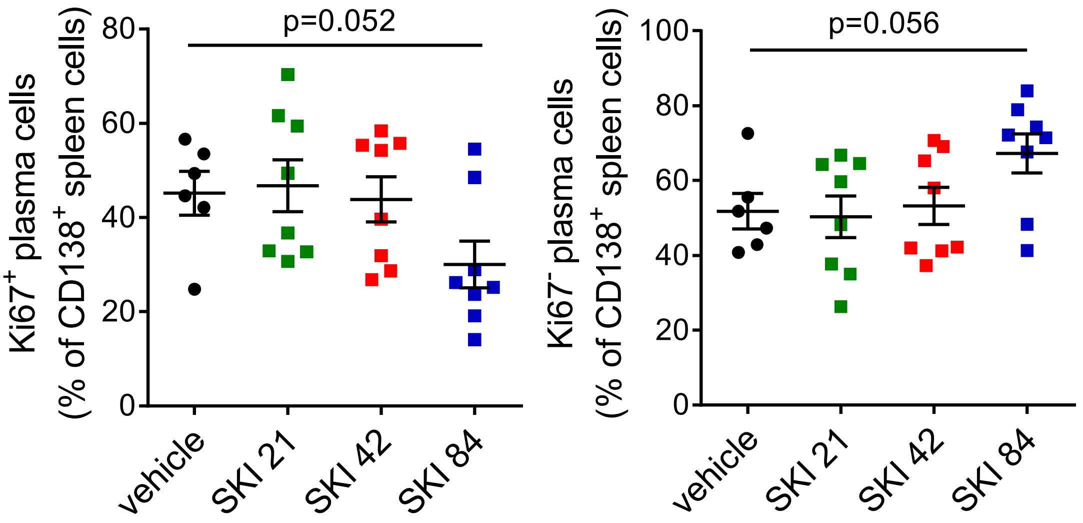

Supplementary Figure 4

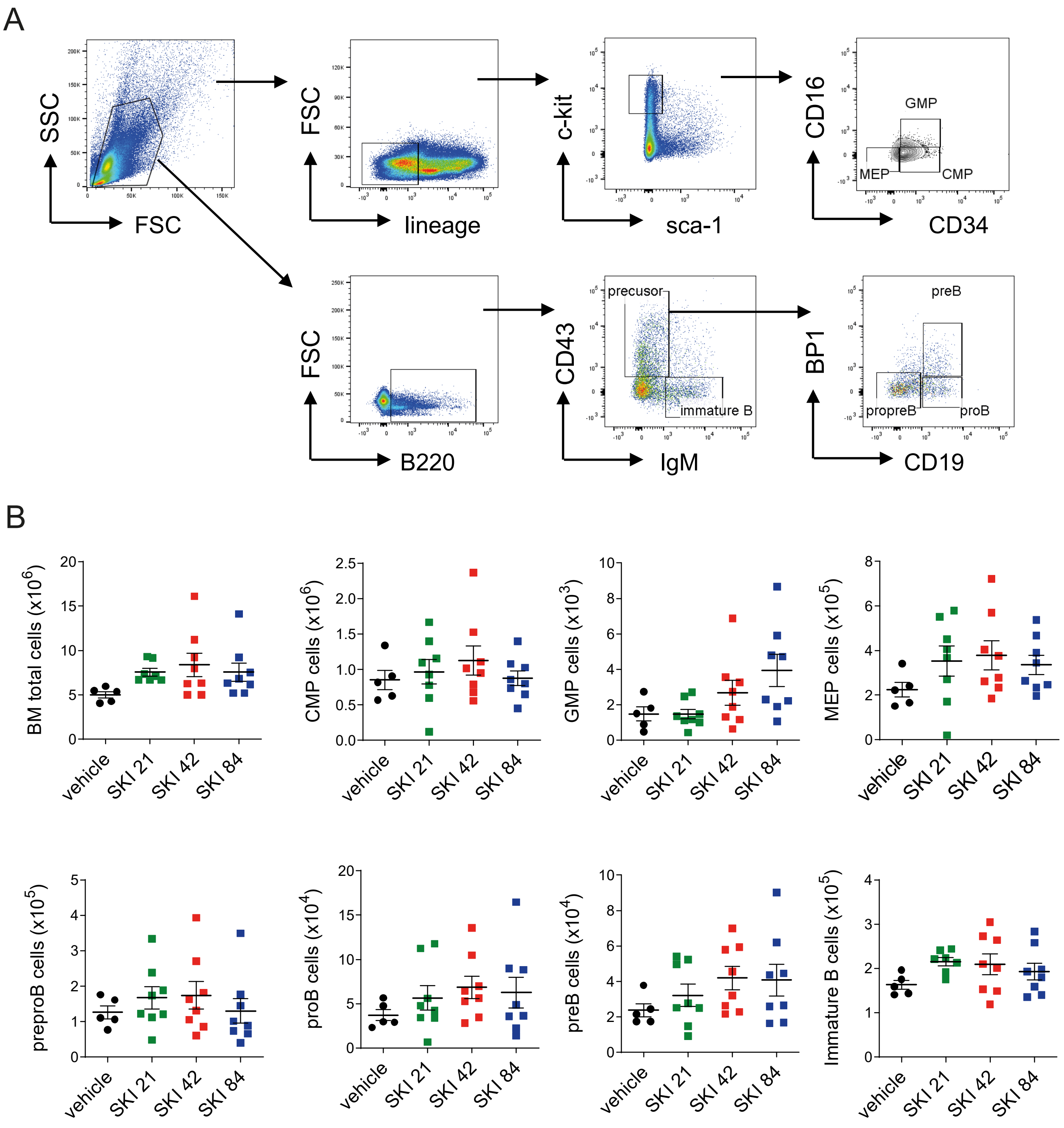

Supplementary Figure 5

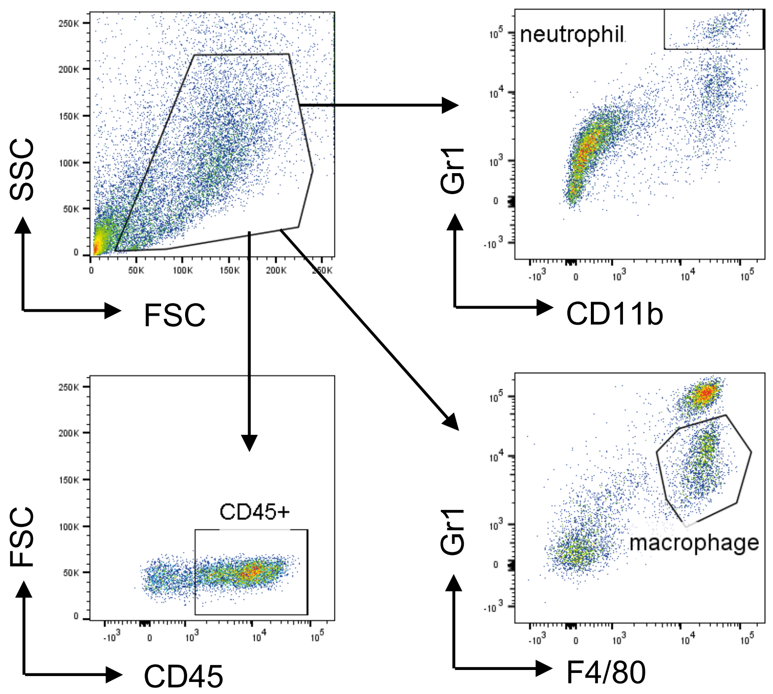

Supplement: uxac096_suppl_Supplementary_Data [file uxac096_suppl_supplementary_data.pdf]
